# Our Response in the Aftermath of the Great Tohoku-Kanto Earthquake and Tsunami

**DOI:** 10.2188/jea.JE20110064

**Published:** 2011-07-05

**Authors:** Suminori Akiba

**Keywords:** earthquake, tsunami, aftermath

First of all, I would like to extend our sincere condolences to the people who suffered from the Great Tohoku-Kanto Earthquake and Tsunami.

In the aftermath of the damage caused by the earthquake and tsunami, the Board of Directors of the Japan Epidemiological Association (JEA) conducted the following activities to provide our colleagues and fellow citizens with useful information that will assist them in coping with the disaster and accurately assessing the health effects of radiation exposure:

(1) Tips and guidelines for coping with the effects of the earthquake and tsunami, as well as information from relevant websites, were posted on our website and sent to our members by email.
(2) On March 25, the Board of Directors made a public announcement on our website entitled “Possible health effects of the Fukushima nuclear accident”, which explained the health effects of radiation exposure, based on information contained in the reports of UNSCEAR (United Nation Scientific Committee on the Effects of Atomic Radiation), and presented our understanding of the situation.
(3) Questions and Answers—a revision of the explanation in our initial announcement, with additional explanatory information, was posted on our website on 13 April.

The direct and indirect effects of the quake and tsunami are enormous, and our society has been seriously affected by this disaster. It will be remembered as a turning point in the history of Japan. For the JEA, it is important to develop and conduct activities that address the consequent health and social problems. As our first academic activity, we plan to hold a symposium related to these concerns at the 22nd Annual Meeting of the JEA, which is being organized by Professor Naohito Yamaguchi. The themes and topics to be addressed are now being discussed by the Academic Activities Committee.

The Japanese government is now making plans to initiate periodical health check-ups and long-term follow-up of residents in Iwate, Miyagi, and Fukushima, the prefectures most affected by the quake and tsunami. There are also plans for similar programs and epidemiological studies of residents evacuated from designated areas and workers who have been mobilized to cope with the nuclear accidents. A number of JEA members are expected to participate in these endeavors, and the JEA will discuss how to provide necessary support for those efforts.

Last, but not least, we offer our thanks for the condolences and support extended by our foreign colleagues.

## POSSIBLE HEALTH EFFECTS OF THE FUKUSHIMA NUCLEAR ACCIDENT

March 25, 2011

Board of Directors

Japan Epidemiological Association

First, we would like to extend our sincere condolences to all who suffered in the Great Tohoku-Kanto Earthquake and Tsunami. As reported by the media, the Japanese government has declared Nuclear Emergency Situations at Fukushima Dai-ichi Nuclear Power Plant (Fukushima I NPP) and Fukushima Dai-ni Nuclear Power Plant (Fukushima II NPP) in accordance with the Act on Special Measures Concerning Nuclear Emergency Preparedness. The spread of radioactive nuclides near the damaged power plants is of great concern. To minimize radiation exposure, residents in a 20-kilometer radius have been instructed to evacuate and those inside a 30-kilometer zone have been advised to remain indoors.

As specialists in epidemiology—an important auxiliary branch of medicine that, among other activities, evaluates the health effects of radiation—we would like to briefly describe the effects of ionizing radiation on human health, based on information contained in the reports of the United Nations Scientific Committee on Atomic Radiation (UNSCEAR), and offer our interpretation. Additional relevant information and links to useful websites are included on the website of the Japan Epidemiological Association (JEA).

### 1. Environmental radiation levels

In this nuclear accident, radioactive nuclides, including iodine-131 (I-131) and Cesium-137 (Cs-137) from the nuclear reactors, were released into the air and spread over wide areas around the plant. The radiation dose decreases rapidly as the distance from the power plant increases. The dose is also affected by wind direction, topography, and the amount of precipitation. Therefore, environmental radiation levels are affected by these factors.

Monitoring data from the Ministry of Education, Science and Technology (MEXT; http://www.mext.go.jp/) indicate that in some areas radiation levels reached several hundred microsieverts per hour (µSv/hr, a common measure of radiation exposure). However, these high radiation levels lasted only for a short period, and the average doses were low.

Radionuclides, including I-131, were found outside of Fukushima Prefecture, and contamination of water, some vegetables, and milk were reported. In addition, a survey by MEXT showed extremely high levels of Cs-137 in soil collected in Fukushima Prefecture, raising concerns about contamination of vegetables. On March 23, I-131 was found in the tap water of the Tokyo metropolitan area and Ibaragi Prefecture, in addition to Fukushima Prefecture. On the basis of these findings, the media recommended refraining from drinking tap water.

The website of the Ministry of Health, Labor and Welfare (MHLW) presents a notice entitled “Measures for tap water if radiation levels exceed safety limits due to the nuclear power plant accident (Water Supply Division, Health Services Bureau)”. It provides the following guidance to heads of departments in charge of water supply administration in each prefecture:

(1) the public should refrain from drinking tap water that exceeds radiation safety limits;
(2) tap water is safe for household use;
(3) tap water intake should not be limited when an alternative source of water is unavailable.

Experts point out that boiling tap water is unlikely to reduce its content of radioactive iodine.

### 2. Effects of radiation exposure

#### (1) Health effects of radiation exposure

The cumulative radiation dose is obtained by multiplying radiation dose per unit by time. The website of the Cabinet Office of the Government of Japan explains it as follows: even if a person remains for six hours in an area where the radiation level is 100 µSv/hr, the cumulative radiation dose is only 600 µSv/hr. In comparison, we are exposed to 2400 µSv of radiation per year from natural sources of radiation, including radionuclides in the environment and cosmic rays, according to a 2008 UNSCEAR report.^*1^ The MEXT website, which presents data from monitoring posts outside the 20-kilometer exclusion zone around the Fukushima I nuclear power plant, also gives examples of typical radiation doses from various everyday sources.

The radiation levels detected by the monitoring posts were low, as were concentrations of radionuclides. Therefore, as long as the current situation does not materially change, the cumulative dose is so low that acute radiation effects will not develop even if residents are exposed to radiation. In addition, the possibility of late effects, which appear after many years, is negligible when compared to differences in health risks among individuals with different lifestyles. Needless to say, there is no threat of radiation exposure from housing evacuees.

The websites of the National Institute of Radiological Sciences and Radiation Effects Association present detailed information on the health effects of radiation. Studies of atomic bomb survivors in Hiroshima and Nagasaki indicate that a single exposure to 1 million µSv (= 1 Sv) of radiation increases cancer risk by 60%. This increase in risk is similar to that observed between nonsmokers and smokers. The effects of radiation exposures that residents have received to date as a result of the Fukushima power plant accident are expected to be much lower.^*2^

#### (2) Effect on the thyroid gland

Exposure to radionuclides such as radioactive I-131 through respiration and intake of water and food is of considerable concern. This is referred to as internal exposure, and radioactive iodines absorbed by the thyroid and its neighboring tissue can damage the thyroid gland. Because the half-life of I-131 is 8 days, the radiation exposure decreases day by day even if the radionuclides are inside the body. The most worrisome health effect of such radiation is thyroid cancer. Fortunately, thyroid cancer is usually not aggressive, and the survival rate is much higher than that of most cancers (http://ganjoho.jp/public/index.html).

Administration of stable non-radioactive iodine is useful for preventing the effects of radioactive iodine. However, it should be carefully administered and expert advice is strongly recommended. Additional information is available in the document entitled “Understanding administration of stable iodine during nuclear emergencies” prepared by the Nuclear Safety Commission of Japan (http://www.nsc.go.jp/bousai/page3/houkoku02.pdf).

Radiation doses below 100 000–200 000 µSv are usually regarded as low. Exposure to radiation from a source outside the body is called external exposure. External exposures at doses higher than the low-dose range increase the risk of thyroid cancer. However, this increase is mainly limited to children, and there is no clear evidence of increased risk among adults. It should also be noted that the effect of internal exposure is less well understood than that of external exposure. The 2006 UNSCEAR report indicated that “in the last few years, information about ^131^I exposures has improved; however, the thyroid cancer risk from ^131^I exposure is still not adequately quantified.”^*3^

#### (3) Effects of low-dose radiation exposure

The present accident resulted in internal exposure from radioactive iodines and other radionuclides, as well as external exposures. However, as of March 24, it is expected that the resident exposure will remain in the low-dose range. It should be noted, however, that continuous monitoring is necessary. Low-dose radiation exposure does not cause acute health effects. There have been many epidemiological studies of the late effects, including cancer, in Japan and other countries. However, none has shown clear evidence that low-dose radiation exposure increases the risk of cancer or other diseases.

### 3. Concluding remarks

To evaluate the health effects of radiation, it is necessary to conduct epidemiological studies and obtain accurate data on humans. However, accurate evaluation of low-dose radiation exposure requires monitoring 100 000 or more people for at least 10 years. For some dose distributions, an even larger study might be necessary. Therefore, conducting an epidemiological study to evaluate the effects of low-dose exposure is not a simple task. To date, such studies have produced no clear evidence regarding the health effects of low-dose radiation exposure.^*4^ To reach a definitive conclusion, it will be necessary to continue or expand ongoing studies or initiate new studies and collect information for extensive evaluation.

An accurate understanding of the effects of radiation on humans is necessary to develop and implement countermeasures to minimize the health effects that might be associated with radiation exposure. If exposed persons have exaggerated ideas of the health effects of radiation, they might suffer undue distress due to their unnecessary concern. In addition, if residents do not understand the effects of radiation, others in the area affected by a nuclear disaster might be treated unfairly or ostracized.

To avoid such situations and reassure residents, it will be necessary to conduct continuous health check-ups of those exposed to radiation. Finally, the Japan Epidemiological Association plans to educate citizens on the effects of radiation by providing them with relevant information and by collaborating with future epidemiological studies.

*1

The 2006 UNSCEAR report estimated common sources of and exposure to natural radiation, as follows:

Direct ionizing and photon component: 280 µSv
Neutron component: 100 µSv
Cosmogenic radionuclides: 10 µSv
External terrestrial radiation: 480 µSv
Uranium and thorium series: 6 µSv
Radon (^222^Rn): 1150 µSv
Thoron (^220^Rn): 100 µSv
Ingestion of ^40^K: 170 µSv
Ingestion of uranium and thorium series: 120 µSv
Total: approximately 2400 µSv

*2

This prediction is based on data from the 2006 UNSCEAR report, which relied on a review of results from epidemiological and other relevant studies.

*3

The 2008 UNSCEAR report made the following points:

(1) The thyroid gland is highly sensitive to the carcinogenic effects of external radiation exposure during childhood.
(2) Age at exposure is an important modifier of risk, and a very strong trend of decreasing risk with increasing age at exposure was observed in most studies.
(3) Although thyroid cancer is more frequent among women, sex differences with respect to radiation risk are unclear.
(4) Among persons exposed during childhood, the risks remain elevated throughout life, although some data suggest that the risk begins to decline at about 20 years after exposure.
(5) The carcinogenic effects of ^131^I are less well understood. Most epidemiological studies of medical exposures have shown little risk after exposure to a wide range of dose levels; however, most of these studies were of adult exposures.
(6) A follow-up study of persons who lived near the Hanford nuclear facility in the United States when they were children showed no evidence of an association between ^131^I dose and thyroid cancer risk.
(7) In contrast, results from studies of people exposed as a result of the Chernobyl accident showed that exposure to radioactive iodine during early childhood was significantly linked with the risk of developing thyroid cancer. The risk appeared to be modified by dietary intake of stable iodine. As was the case for data on external low-LET (Linear Energy Transfer) radiation exposure, data from the Chernobyl accident studies suggest that risk decreases with increasing age at exposure.

*4

On the effects of internal exposure from radionuclides, the UNSCEAR 2010 report to the UN General Assembly stated: “Valuable information on the long-term low-dose exposures to internally incorporated radionuclides has been provided by epidemiological studies of the health of workers at the Mayak nuclear complex in the southern Urals of the Russian Federation, and of the population near the Techa River whose exposure was due to radioactive discharges from that facility. Overall, the cancer risk estimates from these studies do not differ significantly from those obtained from the studies of the atomic-bombing survivors in Japan.”

The report also discussed studies conducted in areas with high levels of background radiation in India and China, about which the report stated: “By contrast, studies on human populations living in areas with elevated natural background radiation in China and India do not indicate that radiation at such levels increases the risk of cancer.”

Indeed, in Kerala, India, there are areas where terrestrial gamma radiation levels are as high as 10 000 µSv/yr (thus, over a period of 50 years, the total dose would be 500 000 µSv). In those areas, more than 10 000 people have lived for many generations.

